# Antiviral Effects of Menthol on Coxsackievirus B

**DOI:** 10.3390/v12040373

**Published:** 2020-03-28

**Authors:** David J.R. Taylor, Syed M. Hamid, Allen M. Andres, Hannaneh Saadaeijahromi, Honit Piplani, Juliana F. Germano, Yang Song, Savannah Sawaged, Ralph Feuer, Stephen J. Pandol, Jon Sin

**Affiliations:** 1 The Smidt Heart Institute and the Barbra Streisand Women’s Heart Center, Cedars-Sinai Medical Center, Los Angeles, CA 90048, USAsyed.hamid@cshs.org (S.M.H.); aandres@health.ucsd.edu (A.M.A.); Hannaneh.Saadaeijahromi@csmns.org (H.S.); honit.piplani@cshs.org (H.P.); juliana.germano@cshs.org (J.F.G.); yang.song@cshs.org (Y.S.); savannah.sawaged@cshs.org (S.S.); 2 The Integrated Regenerative Research Institute (IRRI) at San Diego State University, San Diego State University, San Diego, CA 92182, USA; rfeuer@sdsu.edu; 3 Department of Medicine, Cedars-Sinai Medical Center, Los Angeles, CA 90048, USA; stephen.pandol@cshs.org

**Keywords:** mitochondria, coxsackievirus, antiviral, pancreas, menthol

## Abstract

Coxsackievirus B (CVB) is a common human enterovirus that causes systemic infection but specifically replicates to high titers in the pancreas. It was reported that certain viruses induce mitochondrial fission to support infection. We documented that CVB triggers mitochondrial fission and blocking mitochondrial fission limits infection. The transient receptor potential channels have been implicated in regulating mitochondrial dynamics; namely, the heat and capsaicin receptor transient receptor potential cation channel subfamily V member 1 (TRPV1) contributes to mitochondrial depolarization and fission. When we transiently warmed HeLa cells to 39 °C prior to CVB exposure, infection was heightened, whereas cooling cells to 25 °C reduced infection. Inducing “cold” by stimulating transient receptor potential cation channel subfamily M member 8 (TRPM8) with menthol led to reduced infection and also resulted in lower levels of mitochondrial fission during infection. Additionally, menthol stabilized levels of mitochondrial antiviral signaling (MAVS) which is known to be tied to mitochondrial dynamics. Taken together, this highlights a novel pathway wherein CVB relies on TRPV1 to initiate proviral mitochondrial fission, which may contribute to the disruption of antiviral immunity. TRPM8 has been shown to antagonize TRPV1, and thus we hypothesize that stimulating TRPM8 blocks TRPV1-mediated mitochondrial fragmentation following CVB exposure and attenuates infection.

## 1. Introduction

Coxsackievirus B (CVB) is a significant human pathogen that most commonly causes mild self-resolving symptoms such as fever, rash, and upper respiratory illness. In rare cases, the virus can cause a number of severe systemic inflammatory diseases such as meningo-encephalitis and myocarditis. CVB has particularly strong tropism to pancreatic acinar cells and can cause both mild and severe pancreatitis [1,2]. Pancreatitis is marked by premature activation of digestive enzymes which causes destruction of the exocrine pancreas. This leads to acute inflammation, further damaging the tissue. Though acute pancreatitis is often mild, about 20% of acute pancreatitis cases manifest as severe pancreatitis which can lead to systemic inflammation and death in up to 30% of patients [3,4]. Viral pancreatitis is more prevalent in children; however, adults are also susceptible to acute viral pancreatitis [5,6]. CVB infections can result in chronic pancreatitis which is marked by long-term inflammation of the pancreas that progressively worsens over time. Chronic pancreatitis is a premalignant disease and a major risk factor for pancreatic cancer, which has a one-year survival rate of 20% [7]. A five-year study showed that 34% of patients diagnosed with acute, recurrent, or chronic pancreatitis had elevated titers for coxsackievirus antibodies [8]. This suggests that coxsackieviral infections may be a more prevalent cause of pancreatitis than previously thought. Treatment options for pancreatitis primarily focus on mitigating acute symptoms, thus effective interventions for potentially life-threatening viral pancreatitis are limited.

We and others have shown that enteroviruses including CVB induce autophagy during infection in order to support infection [9,10,11]. Autophagy is a degradation pathway where cells package damaged or unneeded proteins into autophagosomes, the contents of which are degraded after fusing with acidic lysosomes [12]. Autophagy is also a mechanism that can degrade faulty mitochondria, where it is referred to as “mitophagy” [13]. This occurs when mitochondrial inner membrane potential is dissipated, and the organelle becomes depolarized. This is a trigger for selective autophagic degradation wherein mitochondrial fission proteins such as DRP1 and MFF mediate mitochondrial fragmentation [13,14]. After faulty mitochondria are segregated from the mitochondrial network, membrane potential continues to decline which stabilizes PINK1 on the outer mitochondrial membrane. This allows for E3 ubiquitin ligases such as parkin to become recruited which ubiquitinate mitochondrial fragments to be trafficked to the autophagosome (herein called the mitophagosome), while healthy mitochondria are rejoined into the mitochondrial network [13]. Several studies showed that impairing mitochondrial fission or promoting mitochondrial fusion suppresses selective mitophagy [13,15,16,17]. It has also been shown that CVB and other picornaviruses activate autophagy and become engulfed in autophagosomes [9,18]. While some intracellular pathogens are eliminated by autophagy, blocking autophagy suppresses CVB infection [18]. We recently reported that CVB is engulfed by mitophagosomes which are expelled from cells as virus-laden extracellular vesicles (EVs) [19]. Indeed, we saw that blocking mitochondrial fission protein DRP1 with either Mdivi-1 or siRNA or impairing mitophagy by silencing the mitophagy adaptor protein optineurin significantly reduced CVB infection. Thus, mitophagy serves as a non-lytic mode of viral dissemination that not only prolongs host cell survival, but also allows nonenveloped CVB to escape the cell in a hijacked membrane that may mask the virus from neutralizing antibodies. 

Other viruses have also been shown to rely on mitochondrial fission and mitophagy to further infection. Hepatitis B virus induces fission and mitophagy as a method to impair antiviral apoptosis [20]. Similar to CVB, the hepatitis C virus relies on fission and mitophagy to promote viral secretion [21]. Though we have reported that CVB induces mitochondrial fission, which promotes the activation of proviral mitophagy, CVB-induced fission by itself may play a broader role in supporting infection. For example, cytomegalovirus has been shown to induce mitochondrial fission via the viral antiapoptotic protein vMIA [22]. This induction of fission is thought to impair the association of mitochondrial antiviral signaling protein (MAVS) and the endoplasmic reticulum (ER)-associated antiviral protein stimulator of interferon genes (STING), which ultimately dampens the RIG-I-like receptor antiviral pathway. In all, these studies demonstrate the importance of considering mitochondrial dynamics when developing possible antiviral strategies.

It is unclear what mechanisms are responsible for CVB-mediated mitochondrial fission. We have found that members of the transient receptor potential (TRP) channels may be important for alterations in mitochondrial dynamics following CVB infection. TRPV1, commonly known as the heat and capsaicin-activated ion channel, has been shown to depolarize mitochondria and trigger mitochondrial fission following activation [23,24,25]. Our preliminary findings suggest that CVB relies on TRPV1-mediated mitochondrial fragmentation, and treating cells with the specific TRPV1 inhibitor, SB-366791, potently reduces CVB infection. Inversely related to TRPV1 is TRPM8, which is known to be the primary TRP channel that is activated by hypothermia and the cooling compound menthol. It has been shown that TRPM8 is a TRPV1 antagonist [26], and we see that subjecting cells to either hypothermia or menthol treatment results in significantly reduced CVB infection. We hypothesize that TRPM8 activation antagonizes TRPV1-mediated proviral mitochondrial fission via inhibition of mitochondrial fission proteins such as DRP1. When we deliver menthol orally to mice that have been infected with CVB, pancreatic viral titers are significantly reduced, and organ inflammation and tissue damage are also blunted. Because menthol is such a common and safe additive to both food and cough medicines, the notion that menthol could have a potent antiviral effect makes it an appealing potential therapeutic for viral diseases.

## 2. Material and Methods

### 2.1. Cell Culture and Treatments

HeLa RW human cervical cancer cells were cultured in Dulbecco’s modified Eagle’s medium (DMEM) (Gibco, Waltham, MA, USA, 11996-073) supplemented with 10% fetal bovine serum (FBS) (Life Technologies, Carlsbad, CA, USA, 16010-159) and antibiotic/antimycotic cocktail (Life Technologies, 15240-062).

SB-366791 (Cayman Chemical, Ann Arbor, MI, USA, 11019) was dissolved in DMSO at a concentration of 10 mM. HeLa cells were treated with 10 µM SB-366791 or equivalent amount DMSO for 24 h prior to infection.

Menthol (Cayman Chemical Company, 25753) was dissolved in ethanol at a concentration of 1 M. HeLa cells were treated with 1 mM menthol or equivalent amount ethanol for one hour prior to infection.

TRPM8 siRNA (Santa Cruz Biotechnology, Dallas, USA, SC-95009) was reconstituted following manufacturer’s protocol. HeLa cells were transfected using Effectene Transfection Reagent (Qiagen, Hilden, Germany, B00118) according to manufacturer’s suggestions for reagent volumes. A total of 72 h following transfection, media was refreshed and cells were immediately infected.

### 2.2. Generation of Coxsackievirus B3 Expressing Enhanced Green Fluorescent Protein

Recombinant coxsackievirus B3 (pMKS1) expressing enhanced green fluorescent protein (EGFP-CVB) was generated as described previously. CVB clone (pH3) was engineered with SfiI restriction site allowing for DNA insertion. EGFP was amplified from expression plasmids with sequence-specific primers that add flanking SfiI sequences. Amplified products were cloned into linearized pMKS1 to create EGFP-CVB plasmid. HeLa RW cells were transfected with EGFP-CVB construct using Lipofectamine 2000 (Thermo Fisher Scientific, Waltham, MA, USA, 12566014). When cells displayed ~50% cytopathic effect, cells were scraped and subjected to three rounds of freeze/thaw cycles. Freeze fractured cells were then centrifuged at 600 × g for 10 min. Clarified supernatant was collected and considered “passage 1” viral stock. “Passage 2” viral stocks were expanded by infecting new HeLa RW cells with “passage 1” and harvesting as described earlier. “Passage 2” viral stocks were used for all experiments in this study.

### 2.3. Western Blots

Cellular whole lysates were harvested by applying RIPA buffer containing protease inhibitor cocktail (Roche, Basel, Switzerland, 05056489001). Protein concentrations were measured using a bicinchoninic acid solution (Sigma-Aldrich, St. Louis, MO, USA, B9643). Equal amounts protein were loaded into 4–20% Tris-Glycine SDS PAGE gels (Life Technologies, EC6025) and transferred to nitrocellulose membranes (VWR, 27376-991). Prior to blocking, membranes were stained with Ponceau S (Sigma-Aldrich, P7170). Membranes were blocked in 5% nonfat dry milk dissolved in tris-buffered saline with 0.1% Tween-20 (TBS-T) for one hour at room temperature and then incubated in primary antibody diluted in 5% nonfat dry milk overnight at 4 °C. Primary antibodies used in this study were as follows: enterovirus VP1 (1:142, Dako, Santa Clara, CA, USA, M706401-1), TRPM8 (1:1000, Novus Biologicals, Littleton, CO, USA, NBP1-97311SS), phospho-DRP1Ser616 (1:1000, Cell Signaling Technology, Danvers, MA, USA, 3455), and MAVS (1:200, Santa Cruz Biotechnology, SC-166583). Membranes were then incubated in either anti-mouse secondary (1:3000, SeraCare, Milford, USA, CN, 5450-0011) or anti-rabbit secondary (1:3000, VWR, 95058-734). After applying enhanced chemiluminescence (ECL) (GE Healthcare, Chicago, IL, USA, RPN2235), membranes were imaged using Bio-Rad Chemidoc XRS+ System (Bio-Rad Laboratories, Hercules, CA, USA). Densitometry was performed using ImageJ software (National Institutes of Health).

### 2.4. Cell Immunostaining

HeLa cells grown on Permanox chamber slides (Thermo Scientific, 177437) were fixed in 4% formaldehyde (Ted Pella, Redding, CA, USA, 18505) diluted in phosphate buffered solution (PBS). Cells were then permeabilized in PBS with 0.25% Triton-X 100 for 10 min, washed in PBS and blocked for one hour in blocking solution (PBS + 0.1% Tween-20 + 1% bovine serum albumin). Cells were then incubated overnight at 4 °C in anti-TOM70 antibody (1:200, Proteintech, Rosemont, IL, USA, 14528-1-AP) diluted in blocking solution. Cells were then washed in PBS and incubated for one hour protected from light in Alexa Fluor 594-conjugated anti-rabbit secondary antibody (1:100, Invitrogen, Carlsbad, CA, USA, A11037) diluted in blocking solution. Cells were then washed, and coverslipped in Vectashield mounting medium (Vector Laboratories, Burlingame, CA, USA, H-1400).

### 2.5. RNA Isolation and Quantitative PCR

RNA isolation was carried out by using TRIsure (Bioline, London, England, BIO-38033) according to the manufacturer’s protocol, which was subsequently reverse transcribed using RevertAid First strand cDNA synthesis kit (ThermoScientific, K1691). qPCR was performed on CFX96 real time PCR system (Qiagen, Germany). Gene expression levels were quantified using ΔΔCt method with 18s rRNA used as normalization control. Following primer pairs were used for the qPCR analysis: IRF3-Forward 5`-AGAGGCTCGTGATGGTCAAG-3`, IRF3-Reverse 5`-AGGTCCACAGTATTCTCCAGG-3`; IRF7-Forward 5`-GCTGGACGTGACCATCATGTA-3`, IRF7-Reverse 5`-GGGCCGTATAGGAACGTGC-3`; 18s rRNA-Forward 5`-GTAACCCGTTGAACCCCATT-3`, 18s rRNA-Reverse 5`-CATCCAATCGGTAGTAGCGC-3`.

### 2.6. Mouse Treatments

Animal ethics: All mouse work adhered to the National Institutes of Health guidelines and was approved by Cedars-Sinai Medical Center’s Institutional Animal Care and Use Committee (IACUC008697, 11 October 2019–30 September 2022). Prior to sacrifice, animals were first anesthetized with isoflurane and underwent cervical dislocation.

Menthol treatments were prepared by dissolving menthol in water containing 0.2% Tween-80 at a concentration of 25 mg/mL. The 10-week-old male C57BL/6 mice were treated with 100 mg/kg menthol or equivalent volume vehicle via oral gavage. At the same time, mice were infected with 107 plaque forming units of EGFP-CVB with DMEM via intraperitoneal (IP) injection. The following day, mice were treated again with 100 mg/kg menthol or vehicle. Two days post-infection, mice were sacrificed and pancreata were harvested. Tissue was either flash frozen for plaque assay or fixed in 4% formaldehyde for histology.

Cerulein (Bachem, Bubendorf, Switzerland, H-3220) was dissolved in water containing 0.1% ammonium hydroxide at a concentration of 1 mg/mL. This was then diluted 1:100 in sterile saline to make working cerulein solution. The 10-week-old male C57BL/6 mice were first treated with menthol. The following day, mice were given additional dose of menthol and began receiving 50 µg/kg working cerulein or equivalent volume sterile saline via intraperitoneal (IP) injection hourly for 7 h (7 injections total). Animals were then sacrificed 1 h after the final injection. Pancreata were harvested and fixed in 4% formaldehyde for histology.

### 2.7. Plaque Assays

Frozen pancreatic tissue was weighed and then homogenized in DMEM using a TissueLyzer LT instrument (Qiagen, Hilden, Germany). Homogenates were then clarified by centrifuging at 1000 × *g* for 10 min at 4 °C. Plaque assays on pancreatic homogenates were performed as previously described [27]. Briefly, HeLa cells were grown to confluency in 6 well plates. Media were removed from cells, and 400 µL serially diluted pancreas sample was added on top of cells. After one hour of incubation with occasional rocking, infected cells were overlain with 4 mL 50:50 mixture of 1.2% molten agar combined with 2× DMEM. Plates were then incubated at 37 °C for 48 h and agar plugs were subsequently fixed for 20 min with 2 mL plaque fixative containing 25% acetic acid and 75% methanol. Plugs were removed and fixed cells were stained for one hour with 2.34% crystal violet solution. Cells were then washed and plaques were counted.

### 2.8. Histology

Fixed pancreatic tissue was embedded in paraffin and sectioned into 4 µm-thick sections. Tissue sections were then stained with hematoxylin and eosin. Sections were deparaffinized with xylene and rehydrated in decreasing concentrations of ethanol. Sections were stained with Gill 2 Hematoxylin (Richard-Allan Scientific, San Diego, CA, USA, 72504) and Eosin-Y (Richard-Allan Scientific, 71204) according to manufacturer’s protocols. Sections were dehydrated in increasing concentrations of ethanol, cleared in xylene, dried and coverslipped with Cytoseal Mounting Medium (Richard-Allan Scientific, 48212-187).

## 3. Results

### 3.1. Temperature Significantly Alters CVB Infection

**Figure 1:**
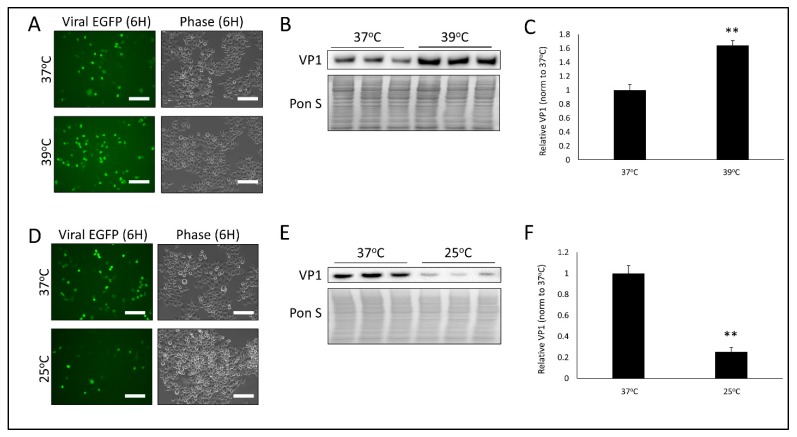
Temperature significantly alters CVB infection- HeLa human cervical cancer cells were kept at either 37 °C, 39 °C or 25 °C for 24 h. Cells were then all incubated at 37 °C for 30 min and infected with enhanced green fluorescent protein coxsackievirus B (EGFP-CVB) at multiplicity of infection of 1 (MOI 1) for 6 h. (**A**) Fluorescence microscopy compares viral EGFP expression between cells kept at either 37 °C or 39 °C. Phase contrast images are shown to the right of respective fluorescence image fields. Scale bars represent 150 µm. (**B**) Western blot on cell lysates from A detecting VP1 viral capsid protein. Ponceau S is shown below. (**C**) Densitometric quantification of western blot in B. (** *p* < 0.01; Student’s t-test, *n* = 3). (**D**) Fluorescence microscopy comparing viral EGFP expression between cells kept at either 37°C or 25 °C. Phase contrast images of same field are shown to the right. Scale bars represent 150 µm. (**E**) Western blot on cell lysates from D detecting VP1 viral capsid protein. Ponceau S is shown below. (**F**) Densitometric quantification of western blot in E. (** *p* < 0.01; Student’s t-test, *n* = 3).

In an earlier report we had demonstrated that CVB infection induces mitochondrial fission, and inhibiting fission significantly impaired infection [19]. Connections between mitochondrial fission and the heat and capsaicin receptor TRPV1 had been documented previously [23,24,25], thus we sought to determine if temperature could influence CVB infection. To test this, we first warmed HeLa human cervical cancer cells to 39 °C for 24 h and then equilibrated cells back to 37 °C for 30 min prior to infecting with CVB expressing enhanced green fluorescent protein (EGFP-CVB) at a multiplicity of infection of 1 (MOI 1). TRPV1 is known to become activated at temperatures above 42 °C [28]; however, we observed that prolonged exposure to 42 °C was cytotoxic to HeLa cells (data not shown). Interestingly, 39 °C has been shown to lead to hypersensitization of TRPV1, [29] therefore we proceeded with this temperature. A total of 6 h post-infection (PI) we found that viral EGFP expression was markedly increased in warmed cells (Figure 1A) and western blots on whole cell lysates revealed significantly increased levels of VP1 viral capsid protein (Figure 1B,C). To test if hypothermic conditions could produce the opposite effect, we cooled cells to 25 °C for 24 h prior to equilibrating to 37 °C for 30 min. After infecting with EGFP-CVB for 6 h, we found that viral EGFP expression was greatly reduced (Figure 1D), as was cellular VP1 levels (Figure 1E,F). These data reflect how profoundly transient hyperthermia and hypothermia can alter CVB infection.

### 3.2. Treatment with TRPV1 Inhibitor SB-366791 or TRPM8 Agonist Menthol Attenuates CVB Infection

**Figure 2:**
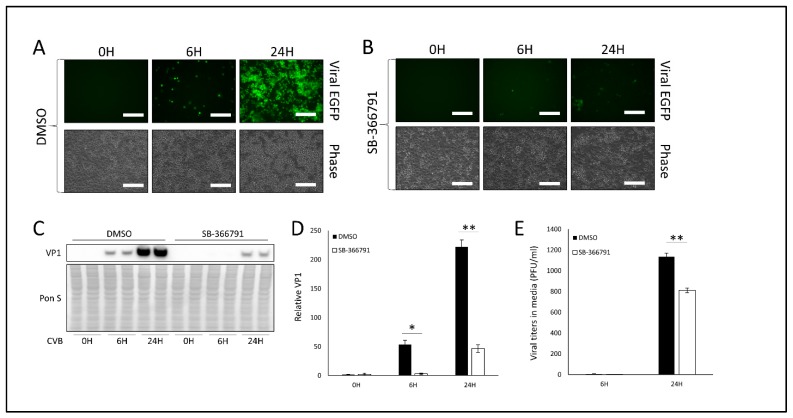
Treatment with TRPV1 inhibitor SB-366791 attenuates CVB infection- HeLa cells were treated with 10 µM of TRPV1 inhibitor SB-366791 or equivalent volume DMSO for 24 h and subsequently infected with EGFP-CVB at MOI 0.1 for 24 h. Fluorescence microscopy compares viral EGFP expression between cells treated with (**A**) DMSO or (**B**) SB-366791. Phase contrast images are shown below respective fluorescence image fields. Scale bars represent 150 µm. (**C**) Western blot on cell lysates from A and B detecting VP1 viral capsid protein. Ponceau S is shown below. (**D**) Densitometric quantification of western blot in C. (** *p* < 0.01; Student’s t-test, *n* = 3). (**E**) Plaque assay quantification of infectious virus in media from cells in A and B. (** *p* < 0.01; Student’s t-test, *n* = 3).

Because high temperature facilitates TRPV1 activation, and we saw that transient hyperthermia amplified CVB infection, we investigated whether treating cells with the selective TRPV1 inhibitor SB-366791 would alter infection. We gave HeLa cells 10 μM SB-366791 for 24 h prior to infecting with EGFP-CVB at MOI 0.1. This dose of SB-366791 did not cause any noticeable cell death after 48 h of treatment (Figure S1A). After 24 h of infection, we found that viral EGFP expression was dramatically blunted (Figure 2A,B). Western blots showed significantly reduced VP1 protein in SB-366791-treated cells at 6 h and 24 h PI as well (Figure 2C,D). Consistent with these findings, plaque assays on cell media revealed a significant reduction in extracellular viral titers at 24 h PI (Figure 2E). Taken together with our earlier hyperthermia study, these data suggest that CVB relies on TRPV1 activity for efficient infection.

**Figure 3:**
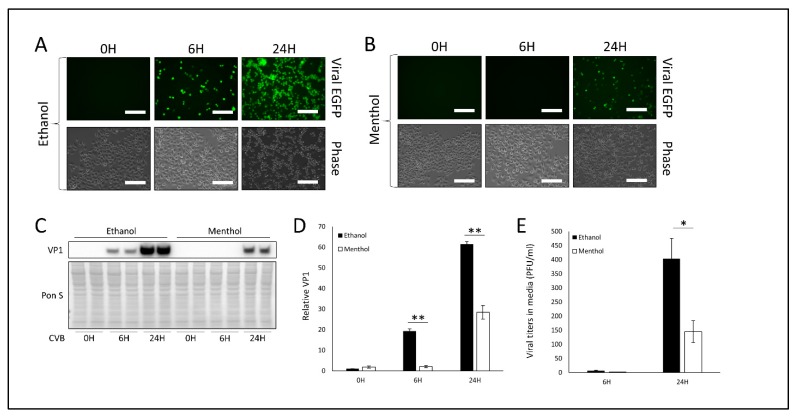
Treatment with TRPM8 agonist menthol impairs CVB infection- HeLa cells were treated with 1 mM menthol or equivalent volume ethanol for 1 h and subsequently infected with EGFP-CVB at MOI 0.1 for 24 h. Fluorescence microscopy compares viral EGFP expression between cells treated with (**A**) ethanol or (**B**) menthol. Phase contrast images are shown below respective fluorescence image fields. Scale bars represent 150 µm. (**C**) Western blot on cell lysates from A and B detecting VP1 viral capsid protein. Ponceau S is shown below. (**D**) Densitometric quantification of western blot in C. (** *p* < 0.01; Student’s t-test, *n* = 3). (**E**) Plaque assay quantification of infectious virus in media from cells in A and B. (* *p* < 0.05; Student’s t-test, *n* = 3).

We next sought to pharmacologically simulate “cold” using the cooling compound menthol. Menthol functions by activating the cold receptor TRPM8 which itself antagonizes TRPV1. We treated HeLa cells with 1 mM menthol for 1 h prior to infection with EGFP-CVB at MOI 0.1. This dose of menthol did not cause any observable cell death after 24 h of treatment (Figure S1B). At 24 h PI, menthol-treated cells expressing viral EGFP were sparse compared to vehicle-treated infected cells (Figure 3A,B). Consistently, western blots on cell lysates showed that VP1 was also markedly reduced at 24 h PI when cells were treated with menthol prior to infection (Figure 3C,D). Plaque assays on cell media similarly showed a significant reduction in extracellular infectious virus in the media from menthol-treated cells (Figure 3E). Thus, TRPM8 activation by either cold or menthol appears to drastically attenuate infection, and this may be a product of TRPV1 antagonism.

### 3.3. Silencing TRPM8 Bolsters CVB Infection

**Figure 4:**
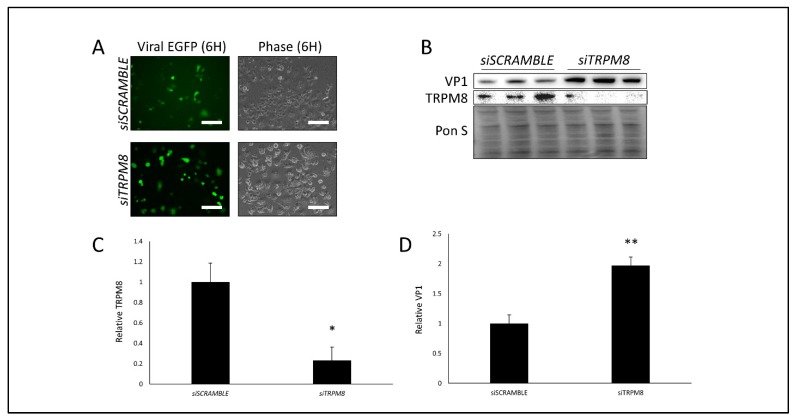
Silencing TRPM8 bolsters CVB infection- HeLa cells were transfected with siRNA targeting TRPM8 (siTRPM8) or scrambled RNA (siSCRAMBLE) for 72 h and subsequently infected with EGFP-CVB at MOI 1 for 6 h. (**A**) Fluorescence microscopy comparing viral EGFP expression between cells transfected with either siSCRAMBLE or siTRPM8. Phase contrast images of same fluorescence image fields are shown to the right. Scale bars represent 150 µm. (**B**) Western blot on cell lysates from A detecting TRPM8 and VP1 viral capsid protein. Ponceau S is shown below. (**C**) Densitometric quantification of TRPM8 western blot in B. (* *p* < 0.05; Student’s t-test, *n* = 3). (**D**) Densitometric quantification of VP1 western blot in B. (** *p* < 0.01; Student’s t-test, *n* = 3).

Our findings thus far demonstrate that cold and menthol can drastically attenuate CVB infection. We hypothesize that this is due to TRPM8 activation which inhibits TRPV1-mediated proviral mitochondrial fission. To truly ascertain whether TRPM8 can serve as an antiviral protein, we next transfected cells with a TRPM8-directed siRNA. Following 72 h of silencing we did not observe any differences in cell viability (Figure S1C) and TRPM8 levels were significantly reduced as determined by western blot (Figure 4B,C). After infecting with EGFP-CVB at MOI 1, we found that at 6 h PI, viral EGFP expression was increased when TRPM8 was suppressed (Figure 4A), and western blots revealed that VP1 viral capsid protein was significantly elevated as well (Figure 4B,D). These data are consistent with our observations with menthol treatment and further support that TRPM8 may have a role in viral suppression.

### 3.4. Menthol Treatment Reduces Mitochondrial Fission Basally and During CVB Infection

**Figure 5:**
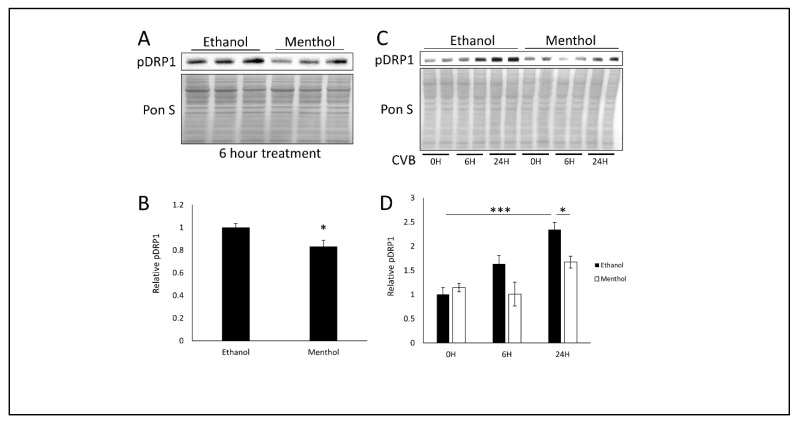
Menthol treatment reduces mitochondrial fission basally and during CVB infection- HeLa cells were treated with 1 mM menthol and then examined after 6 h. (**A**) Western blot on cell lysates detecting mitochondrial fission marker phospho-DRP1Ser616 (pDRP1). Ponceau S is shown below. (**B**) Densitometric quantification of western blot in A. HeLa cells were treated with 1 mM menthol or equivalent dose of ethanol for 1 h and subsequently infected with EGFP-CVB at MOI 0.1 for 24 h. (**C**) Western blot on cell lysates detecting pDRP1. Ponceau S is shown below. (**D**) Densitometric quantification of western blot in C. (* *p* < 0.05, *** *p* < 0.001; Student’s t-test, *n* = 3–4).

As mentioned previously, TRPV1 has been implicated in the regulation of mitochondrial dynamics, specifically that TRPV1 activation can lead to mitochondrial depolarization and subsequent mitochondrial fission [23,24,25]. We had reported that CVB-mediated mitochondrial fission leads to enhanced infection, and suppressing fission via DRP1 inhibition significantly blunts infection [19]. Because menthol activates TRPM8, which in turn inhibits TRPV1, we examined if menthol treatment would alter mitochondrial morphology. We treated HeLa cells with menthol for 6 h prior to staining for outer mitochondrial membrane marker TOM70. Whereas vehicle treated HeLa cells possessed short tubular mitochondrial networks, menthol-treated HeLa cells possessed very long mitochondrial filaments, which may be a result of reduced basal mitochondrial fission (Figure S2). Indeed after 6 h of menthol treatment, western blots revealed that mitochondrial fission marker phospho-DRP1Ser616 (pDRP1) was also significantly reduced (Figure 5A,B), which supports a bias towards mitochondrial interconnectivity. When examining pDRP1 levels during the course of infection, we see that menthol treatment causes a significant reduction in CVB-mediated mitochondrial fission (Figure 5C,D). This appears to be a mechanism by which menthol can impede CVB infection as it recapitulates our previously reported findings that inhibiting mitochondrial fission potently suppresses CVB infection [19].

### 3.5. Menthol Enhances Antiviral Immunity During Infection

**Figure 6:**
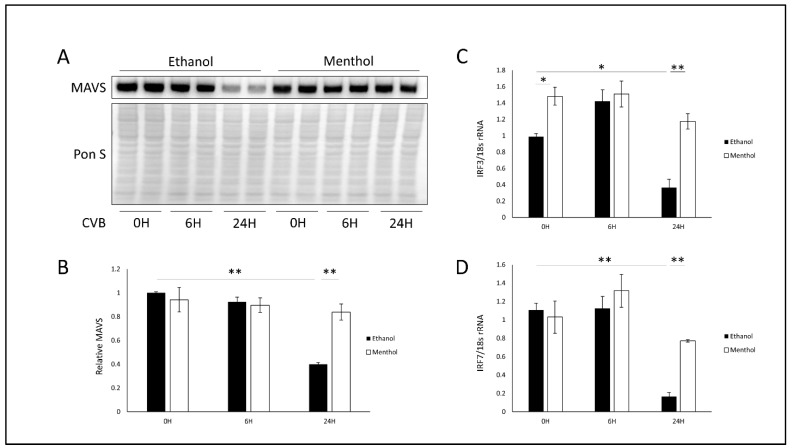
Menthol enhances antiviral immunity during infection- HeLa cells were treated with 1 mM menthol or equivalent dose ethanol for 1 h and subsequently infected with EGFP-CVB at MOI 0.1 for 24 h. (**A**) Western blot on cell lysates detecting mitochondrial antiviral signaling (MAVS). Ponceau S is shown below. (**B**) Densitometric quantification of western blot in A. (** *p* < 0.01; Student’s t-test, *n* = 3). mRNA was extracted from these cells and quantitative PCR was performed to detect (**C**) IRF3 or (**D**) IRF7. (* *p* < 0.05, ** *p* < 0.01; Student’s t-test, *n* = 3).

Virus-induced mitochondrial fission has been shown to disrupt mitochondria-based antiviral signaling [22]. CVB has been shown to both activate DRP1-mediated mitochondrial fission and abrogate MAVS [19,30,31]. We tested whether the addition of menthol altered MAVS expression during the course of CVB infection. We pretreated HeLa cells with menthol and infected with EGFP-CVB at MOI 0.1. As expected, we found that in vehicle-treated infected cells, MAVS levels were significantly attenuated at 24 h of infections (Figure 6A,B). Interestingly, menthol-treated infected cells retained high levels of MAVS expression, which may contribute to the antiviral properties of menthol. Indeed, quantitative PCR revealed that interferon regulatory factor 3 (IRF3) and IRF7, which are antiviral proteins activated downstream of MAVS, were expressed at significantly higher levels at 24 h PI in menthol-treated cells compared to vehicle-treated cells (Figure 6C,D). Taken together, our data suggest that menthol may provide antiviral effects by preventing virus-induced mitochondrial fission and thus preserving cellular antiviral immunity.

### 3.6. Oral Menthol Treatment Reduces Pancreatic CVB Titers and Tissue Destruction in CVB-Infected Mice

**Figure 7:**
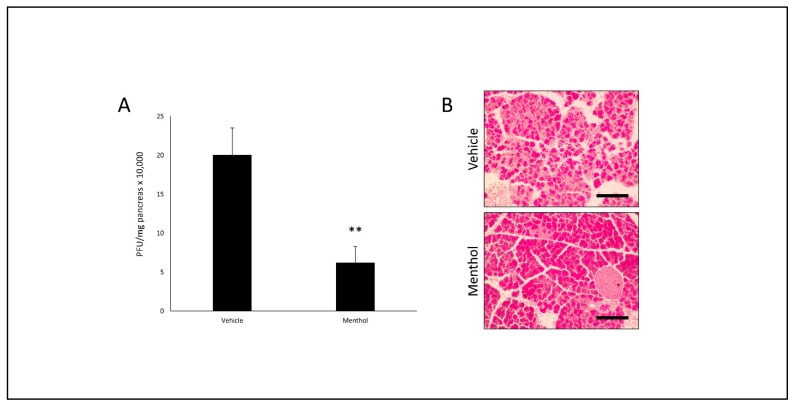
Oral menthol treatment reduces pancreatic CVB titers and tissue destruction in CVB-infected mice- The 10-week-old C57BL/6 male mice were treated with 100 mg/kg menthol or equivalent volume vehicle each day for 2 days via oral gavage. At the same time as the first menthol dose, mice were infected IP with 107 plaque forming units (PFU) EGFP-CVB. A total of 2 days post-infection (PI), mice were sacrificed, and pancreata were assessed. (**A**) Pancreatic viral titers as measured by plaque assays on pancreatic homogenates. (** *p* < 0.01; Student’s t-test, *n* = 6–7). (**B**) Hematoxylin and eosin staining on representative pancreas sections. Scale bars represent 150 µm.

To test the efficacy of menthol at suppressing CVB infection in vivo we infected C57BL/6 mice with 107 EGFP-CVB via IP injection. We have seen that this dose of CVB can potently cause pancreatic inflammation and necrosis as early as 2 days PI, however animals do not display any overt symptoms such as moribundity or lack of feeding and grooming. At the same time as infection, we began treating mice with 100 mg/kg daily with menthol or equivalent volume vehicle via oral gavage. A total of 2 days PI, we harvested mouse pancreata to assess infection. Plaque assays revealed that oral menthol dosing significantly reduced pancreatic viral titers by roughly 69% (Figure 7A). Hematoxylin and eosin staining showed that pancreatic tissue from vehicle-treated infected animals contained large areas of immune cell infiltration indicating severe inflammation, interstitial spacing between acini as a result of pancreatic edema, and sparse eosin staining due to necrosis (Figure 7B, Figure S3). Menthol-treated animals also exhibited pancreatic damage; however, the severity was generally markedly attenuated compared to vehicle controls. These results recapitulate our in vitro observation demonstrating the antiviral potency of menthol.

TRPV1 has been implicated in the context of pancreatitis. Studies have shown that TRPV1 transcripts are elevated in experimental non-infection animal models of pancreatitis [32,33]. This elevation in TRPV1 activity contributes to neurogenic pancreatic inflammation, and treatment with TRPV1 antagonists reduces the severity of disease [34]. To test if the effects of menthol could be attributed to suppression of inflammation rather than antiviral activity, we tested whether menthol treatment could limit the onset of cerulein-induced pancreatitis. Hourly 50 µg/h cerulein injections caused pancreatitis to manifest rapidly within 7 h [35], therefore we pretreated mice with menthol 1 day prior to cerulein exposure. At the start of the cerulein regimen, we treated mice an additional dose of menthol. After the conclusion of cerulein treatment, we found that both vehicle-treated and menthol-treated animals exhibited similar degrees of pancreatic tissue destruction (Figure S4). These data indicate that the protection menthol confers during viral infection is associated more with antiviral effects rather than specific anti-inflammatory qualities.

## 4. Discussion

**Figure 8:**
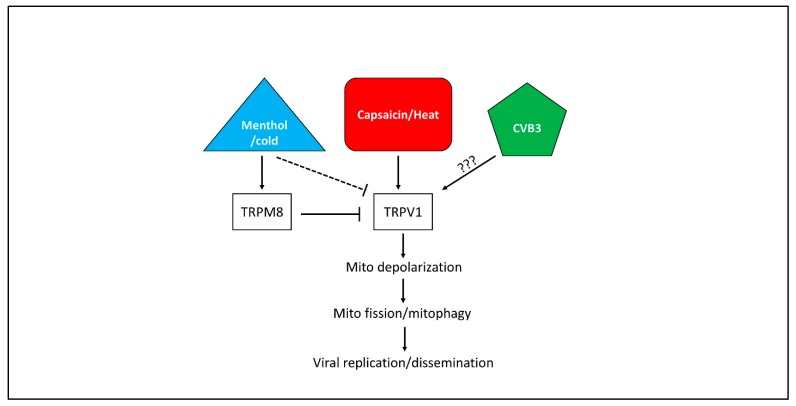
Hypothetical model of TRP channels and CVB infection- CVB has previously been shown to rely on mitochondrial fission and mitophagy to promote infection. Activity of the capsaicin and heat receptor TRPV1 appears to be important for CVB infection, and this may be due to its ability to cause mitochondrial depolarization and subsequent mitochondrial fission. It is unclear if CVB itself upregulates TRPV1 activity. The cold and menthol receptor TRPM8 antagonize TRPV1, thus menthol treatment indirectly suppresses TRPV1-mediated mitochondrial fission and blunts CVB infection.

To date, few reports have established a connection between TRP channels and viral infections, and none have associated TRP channels with coxsackieviral infections. TRP channels have primarily been characterized in infections with respiratory viruses such as rhinovirus, measles, and respiratory syncytial virus which have been shown to upregulate TRPV1 causing hypersensitization to cough and promotion of airborne viral dissemination [36]. A 2003 study demonstrated that menthol has direct virucidal effects on herpes simplex virus 1 and 2 [37]. The authors attributed this to possible interference with the viral envelope or disruption of viral attachment to host cells. It is unclear if any of these aspects apply to non-enveloped viruses such as CVB. Because TRPV1 inhibition via SB-366791 significantly limits CVB infection, we hypothesize that TRPV1 stimulators such as heat or capsaicin amplify infection by inducing mitochondrial depolarization which leads to mitochondrial fission (Figure 8). Not only could this support proviral mitophagy which we described recently, [19] it could also potentially disrupt mitochondria-based antiviral machinery which was observed in the context of cytomegaloviral infection [22]. Menthol and cold treatment indirectly inhibits TRPV1 activity by stimulating its antagonist, TRPM8. Indeed, we observed that treating HeLa cells with menthol reduced mitochondrial fission and led to formation of long filamentous mitochondrial networks. It is unclear if TRPM8 alters mitochondrial morphology directly or if it only does this by inhibiting TRPV1. Additionally, it is unknown if CVB infection itself upregulates TRPV1 activity, as is the case with a number of other respiratory viruses

We associate the antiviral effects of menthol with its ability to impair mitochondrial fission. In one of our earlier reports we showed that during CVB infection, the virus localizes to mitochondria, induces DRP1-mediated mitochondrial fission, and causes the autophagic engulfment of virus-laden mitochondria [19]. These infectious mitophagosomes become released from the infected cell as a nonlytic mode of viral dissemination. We had also observed that these structures may contain proviral microRNA which amplifies downstream infection [38]. Inhibiting mitochondrial fission by silencing DRP1 or treating with Mdivi-1 led to significant reductions in CVB infection. We hypothesize that a similar effect occurs with menthol treatment. This is evidenced by reduced phosphorylation of DRP1 and increased presence of long tubular mitochondria following menthol treatment. Additionally, CVB-induced fission by itself may play a broader role in supporting infection. As mentioned, cytomegalovirus has been shown to induce mitochondrial fission via the viral antiapoptotic protein vMIA which ultimately disrupts MAVS-based antiviral signaling [22]. Because MAVS signaling also plays a central part in cellular defense against CVB infections, it is reasonable to believe that CVB-induced mitochondrial fission also impairs antiviral signaling.

Menthol could be an appealing therapeutic when treating CVB infections as it is relatively safe, cost-effective and commonly available. Our data also suggest that oral consumption of menthol could confer potent efficacy in reducing pancreatic infection. It is unclear as yet if oral menthol delivery could provide system-wide antiviral benefit such as in the case of meningitis and myocarditis; however, because the pancreas is a major initial sync for coxsackievirus prior to systemic infection, attenuating viral titers in the pancreas could potentially prevent further viral spread. It has been shown in mice that coxsackieviral myocarditis is preceded by pancreatitis, and if pancreatitis is absent, then infection generally does not progress to myocarditis [1]. Further testing in other CVB disease models as well as with other viruses would greatly elucidate how treatment with menthol or other TRP channel effectors could be used to attenuate viral infections.

## Supplementary Materials

The following are available online at https://www.mdpi.com/1999-4915/12/4/373/s1, Figure S1: Cell viability following SB-366791, menthol, or *siTRPM8* treatment, Figure S2: Menthol causes mitochondrial elongation, Figure S3: Menthol-treated animals exhibit lesser degree of CVB-induced pancreatic damage, Figure S4: Menthol does not affect non-infection cerulein-induced pancreatitis.
