# Supplementary material for: Antiviral Effects of Menthol on Coxsackievirus B
Source: Viruses. 2020 Mar 28;12(4):373. doi: 10.3390/v12040373 (PMC7232514; doi:10.3390/v12040373)
Supplement: Supplementary file 1 [file viruses-12-00373-s001.pdf]

SUPPLEMENTAL FIGURES

Supplemental Figure S1

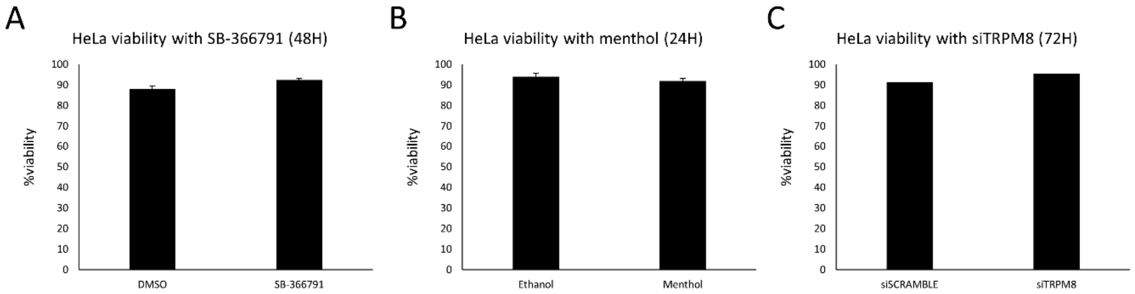

Supplemental Fig S1: Cell viability following SB-366791, menthol, or *siTRPM8* treatment- Trypan blue was used to distinguish dead versus viable cells in HeLa cells treated with either (**A**) 10 µM SB-366791 for 48 hours, (**B**) 1 mM menthol for 24 hours, or (**C**) *siTRPM8* for 72 hours along with respective controls. (Student's t-test where applicable; n=3 for SB-366791 and menthol; n=1 for *siTRPM8*)

Supplemental Figure S2

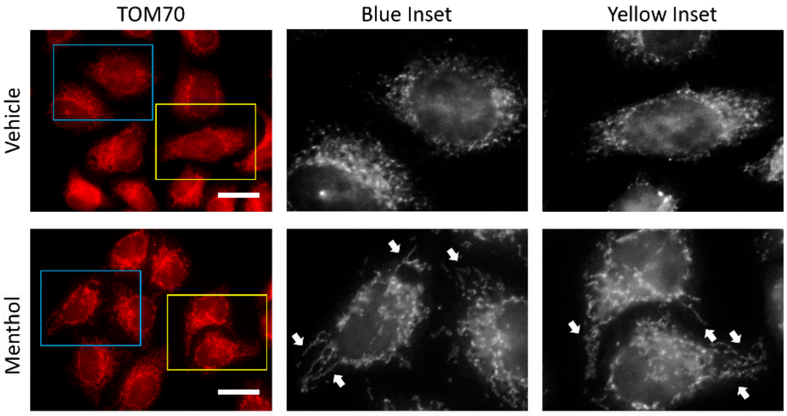

Supplemental Fig S2: Menthol causes mitochondrial elongation- Fluorescence microscopy on cells immunostained for outer mitochondrial membrane marker TOM70. Scale bars represent 25 µm. White arrows indicate long tubular mitochondria.

Supplemental Figure S3

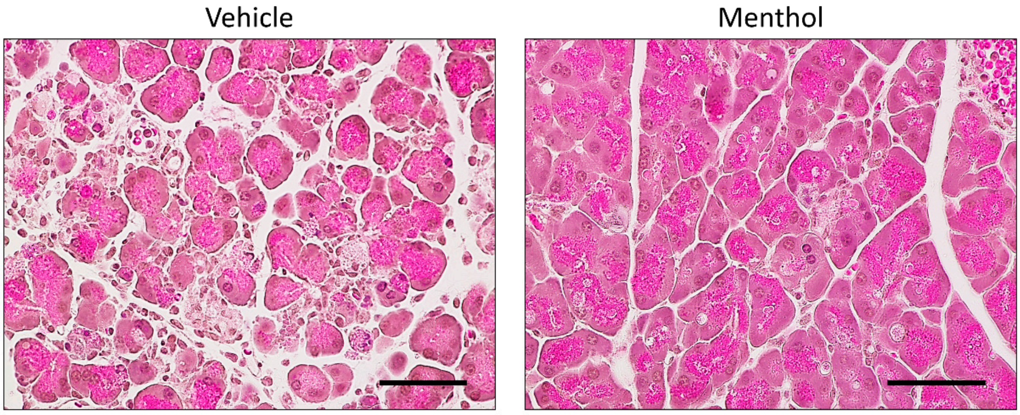

Supplemental Fig S3: Menthol-treated animals exhibit lesser degree of CVB-induced pancreatic damage- Representative high magnification images of hematoxylin and eosin-stained pancreata from animals in **Fig 7**. Scale bars represent 50 µm.

Supplemental Figure S4

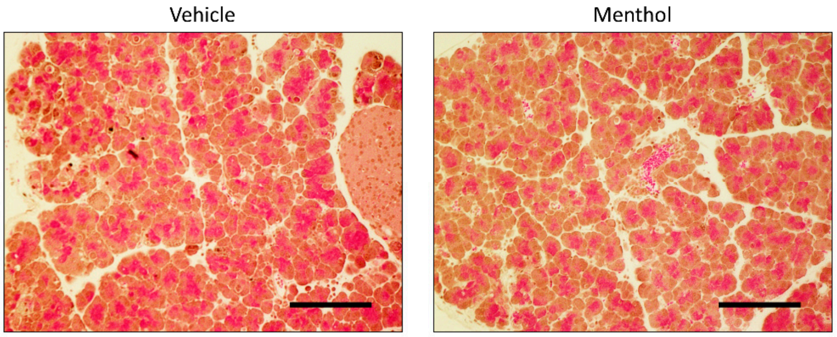

Supplemental Fig S4: Menthol does not affect non-infection cerulein-induced pancreatitis**-** 10 week old C57BL/6 mice were treated with 100 mg/kg menthol or equivalent volume vehicle 1 day prior to cerulein injections. The following day, mice were given an additional dose of menthol or vehicle and began receiving IP injections of 50 µg/kg cerulein or equivalent volume sterile saline hourly for 7 hours (7 injections total). Hematoxylin and eosin staining on representative pancreas sections are shown. Scale bars represent 150 µm.
